# Proteomic analysis of extracellular vesicles reveals an immunogenic cargo in rheumatoid arthritis synovial fluid

**DOI:** 10.1002/cti2.1185

**Published:** 2020-11-07

**Authors:** Andrew D Foers, Laura F Dagley, Simon Chatfield, Andrew I Webb, Lesley Cheng, Andrew F Hill, Ian P Wicks, Ken C Pang

**Affiliations:** ^1^ The Walter and Eliza Hall Institute of Medical Research Parkville VIC Australia; ^2^ Department of Medical Biology University of Melbourne Parkville VIC Australia; ^3^ Department of Rheumatology Royal Melbourne Hospital Parkville VIC Australia; ^4^ Department of Biochemistry and Genetics La Trobe Institute for Molecular Science La Trobe University Bundoora VIC Australia; ^5^ Murdoch Children's Research Institute Parkville VIC Australia; ^6^ Department of Paediatrics University of Melbourne Parkville VIC Australia; ^7^ Department of Adolescent Medicine Royal Children's Hospital. Parkville VIC Australia

**Keywords:** citrullination, extracellular vesicles, neutrophils, osteoarthritis, rheumatoid arthritis, synovial fluid

## Abstract

**Objectives:**

Extracellular vesicles (EVs) from rheumatoid arthritis (RA) synovial fluid (SF) have been reported to stimulate the release of pro‐inflammatory mediators from recipient cells. We recently developed a size exclusion chromatography (SEC)‐based method for EV isolation capable of high‐quality enrichments from human SF. Here, we employed this method to accurately characterise the SF EV proteome and investigate potential contributions to inflammatory pathways in RA.

**Methods:**

Using our SEC‐based approach, SF EVs were purified from the joints of RA patients classified as having high‐level (*n* = 7) or low‐level inflammation (*n* = 5), and from osteoarthritis (OA) patients (*n* = 5). Protein profiles were characterised by mass spectrometry. Potential contributions of EV proteins to pathological pathways and differences in protein expression between disease groups were investigated.

**Results:**

Synovial fluid EVs were present at higher concentrations in RA joints with high‐level inflammation (*P‐*value = 0.004) but were smaller in diameter (*P‐*value = 0.03) than in low‐level inflammation. In total, 1058 SF EV proteins were identified by mass spectrometry analysis. Neutrophil and fibroblast markers were overrepresented in all disease groups. Numerous proteins with potential to modulate inflammatory and immunological processes were detected, including nine citrullinated peptides. Forty‐five and 135 EV‐associated proteins were significantly elevated in RA joints with high‐level inflammation than in RA joints with low‐level inflammation and OA joints, respectively. Gene ontology analysis revealed significant enrichment for proteins associated with ‘neutrophil degranulation’ within SF EVs from RA joints with high‐level inflammation.

**Conclusion:**

Our results provide new information about SF EVs and insight into how EVs might contribute to the perpetuation of RA.

## Graphical abstract

Here, we apply a stringent size‐exclusion chromatography‐based method in combination with quantitative proteomics to accurately profile protein content within synovial fluid extracellular vesicles from a cohort of arthritis patients. Our results show large numbers of extracellular vesicles from neutrophil and fibroblast origin are present in rheumatoid arthritis synovial fluid, and these contain a protein cargo with capacity to stimulate immune responses. Our results provide new information about synovial fluid extracellular vesicles and insight into how extracellular vesicles might contribute to the perpetuation of rheumatoid arthritis.

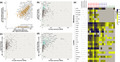

## Introduction

Rheumatoid arthritis (RA) is a chronic, systemic autoimmune disease that targets synovial joints, and can lead to the irreversible destruction of articular cartilage and bone. RA is thought to be because of an abnormal immune response to as yet unknown antigens, with persistent inflammation in affected joints. Although treatment strategies have improved in recent years, RA remains a lifelong affliction. A better understanding of RA pathophysiology is required to advance treatment strategies and improve patient outcomes.

Extracellular vesicles (EVs) are small (40–500 nm) membrane vesicles released from cells. EVs contain a cargo of protein and RNA capable of eliciting responses in recipient cells and carry markers of the cell of origin. By stimulating the release of pro‐inflammatory cytokines, EVs have been proposed to contribute to joint inflammation in RA.^1^, ^2^ For instance, EVs from RA synovial fluid (SF) can induce IL‐6, IL‐8, MCP‐1, RANTES, VEGF and BAFF release from fibroblast‐like synoviocytes (FLS)^3^, ^4^, ^5^ and leukotriene B4 release from neutrophils.^6^ However, EVs have also been proposed to protect against joint destruction. For instance, neutrophil‐derived EVs containing annexin 1 (ANXA1) are reported to promote anabolic activity in recipient chondrocytes^7^ and inhibit inflammatory activation of synovial macrophages.^8^

In this way, the mechanisms by which EVs influence RA pathogenesis remain poorly defined. Moreover, high‐quality EV purifications are vital for accurately identifying EV content and function, and contaminants co‐purifying with SF EVs may have compromised previous investigations. For example, one study identified albumin as the most abundant protein present in EVs isolated from RA SF,^9^ but albumin has since been shown to be a major contaminant in EVs prepared from plasma^10^ and SF^11^ using traditional EV isolation methods.

Previously, we developed a size exclusion chromatography (SEC)‐based method of EV enrichment capable of high‐quality EV purifications from SF.^11^ With the aim of investigating EV content that might be pathogenic in RA, we applied this method, in combination with quantitative proteomics, to profile proteins within SF EVs from a cohort of RA patients. SF EVs were analysed from OA patients as non (or at least less)‐inflammatory controls. Our results provide further support for the possibility of pro‐inflammatory effects from SF EVs in RA and define specific EV proteins likely to be involved in mediating these effects.

## Results

### Characterisation of EV isolation

**Figure 1:**
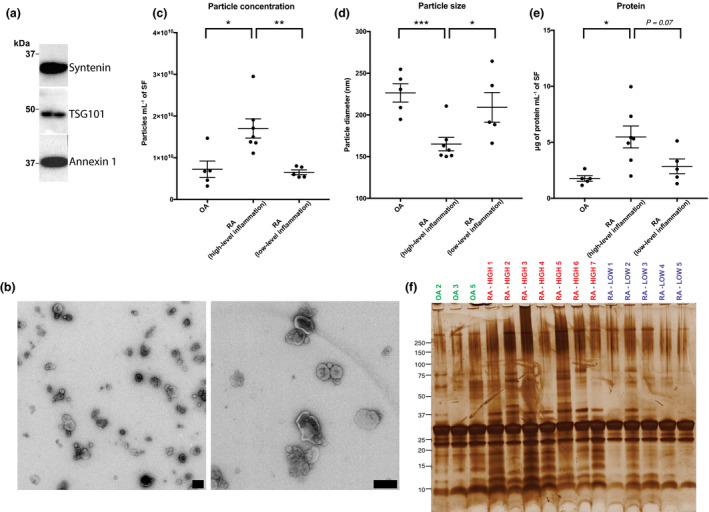
Comparison of synovial fluid (SF) extracellular vesicle (EV) size, diameter and protein content in OA, and rheumatoid arthritis (RA) joints with either high‐ or low‐level inflammation. Proteinase K‐treated size exclusion chromatography EV enrichments were assessed by **(a)** Western blotting and **(b)** transmission electron microscopy. Particle **(c)** concentration and **(d)** diameter in size exclusion chromatography eluents as determined by nanoparticle tracking analysis. **(e)** Comparison of total EV protein concentration in SF from OA, RA (high‐level inflammation) and RA (low‐level inflammation) joints. **(f)** Gel electrophoresis with silver staining of SF EVs isolated from individual patients. **(b)** Scale bars = 200 nm. **(c, d)** Each point represents an average of 5 technical replicates. **(e)** Each point represents an average of 1–2 technical replicates. **(c–e)** OA, *n* = 5; RA (high‐level inflammation), *n* = 7; RA (low‐level inflammation), *n* = 5. Data were analysed with the Student's test. Error bars represent SEM. * denotes *P‐*value < 0.05. ** denotes *P‐*value < 0.01, and *** denotes *P‐*value < 0.001. **(f)** Equal protein mass was loaded for each sample.

To confirm SF EV preparations were of sufficient quality, EV enrichments were evaluated by Western blotting for canonical EV markers syntenin, TSG101 and ANXA1 and transmission electron microscopy. High‐quality enrichments were confirmed by Western blotting (Figure 1a) and transmission electron microscopy, with minimal amounts of non‐EV‐contaminating material (Figure 1b). These observations are consistent with our previous report showing that SEC coupled with proteinase K is a reliable method for obtaining high‐quality EV enrichments from SF.^11^

We next profiled EVs within SF obtained from the joints of RA patients characterised as having either high‐ or low‐level inflammation based on SF white cell counts^12^ and also compared SF from OA patients, which are typically non‐inflammatory. Patient details are summarised in Table 1 with additional detail on individual patient characteristics and background therapies in Supplementary table 1.

**Table 1:** Summary of patient details and clinical parameters

	RA (high‐level inflammation)	RA (low‐level inflammation)	OA	*P*‐value [RA (high‐level inflammation) vs RA (low‐level inflammation)]	*P*‐value [RA (high‐level inflammation) vs OA]	*P*‐value [RA (low‐level inflammation) vs OA]
*N*	7	5	5	–	–	–
Age – mean (s.d.)	65.0 (11.7)	70.2 (9.7)	62.2 (13.5)	0.44	0.71	0.31
Sex – number of females/males	3/4	3/2	4/1	> 0.99	0.29	> 0.99
White cell count – mean (s.d.) cells µL^−1^	10 013 (5610)	259 (241)	340 (212)	*0.003*	*0.004*	0.63
Anti‐citrullinated protein antibody (% positive)	71%	40%	–	0.56	–	–
Rheumatoid factor (% positive)	71%	60%	–	> 0.99	–	–
Disease Activity Score 28 – median (range)	4.35 ( 3.31–5.50)	3.70 (2.60–5.00)	–	0.35	–	–
C‐reactive protein – median (range) mg L^−1^	20 (6–164)	2 (2–26)	–	0.21	*–*	–

Sex, anti‐citrullinated protein antibody, and rheumatoid factor positivity were analysed with Fisher's exact test. All other parameters were analysed with the Student's *t*‐test.

RA, rheumatoid arthritis. Italics indicates a *P*‐value < 0.05.

### SF EVs in highly inflamed RA joints are present at high concentrations and have greater protein diversity

First, differences in SF EV abundance, size and protein profiles between disease groups were investigated. Nanoparticle tracking analysis of SEC eluent identified roughly twice as many particles in SF from RA joints with high‐level inflammation compared to those with low‐level inflammation (*P‐*value = 0.004) and OA (*P‐*value = 0.01; Figure 1c). The average particle size was also significantly lower in highly inflamed RA SF than in SF from RA joints with low‐level inflammation (*P‐*value = 0.03) and OA joints (*P‐*value = 0.001; Figure 1d), suggesting increases of a particular EV subtype in joints with high‐level inflammation. Consistent with elevated numbers of EVs, increases in EV protein concentration per mL of SF were detected in EVs enriched from RA joints with high‐level inflammation (Figure 1e). Distinct protein patterns in SF EVs from RA joints with high‐level inflammation were also observed by gel electrophoresis, indicating greater diversity in the protein profile within these EVs (Figure 1f).

### Protein markers of neutrophil and fibroblast origin are enriched in EVs from RA SF

**Figure 2:**
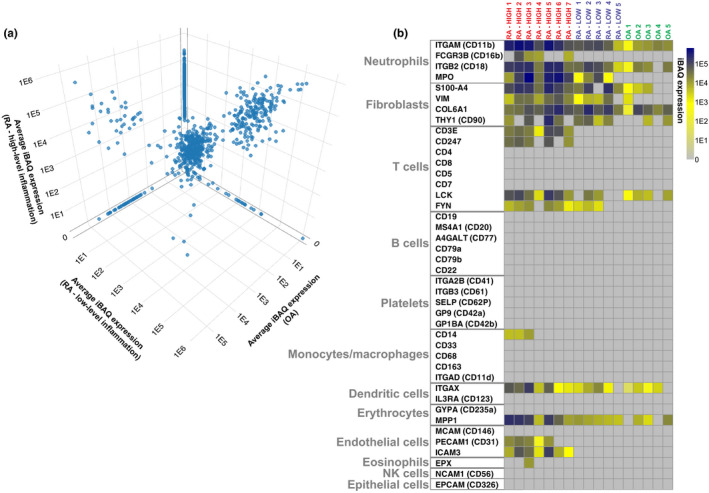
Expression of cellular lineage markers in proteomics dataset. **(a)** 3D scatter plot comparing expression of all 1058 identified proteins across disease groups. **(b)** Expression of specific cellular lineage markers within the proteomics dataset is visualised by heatmap. Heatmap rows show individual lineage markers with originating cell types indicated. Heatmap columns represent individual synovial fluid (SF) extracellular vesicle (EV) samples. Intensity‐based absolute quantification (iBAQ) values are scaled as indicated. Proteins not detected are coloured in grey. UniProt gene names are specified.

Next, protein expression within EVs was quantified by mass spectrometry (MS) analysis. In total, peptides from 1058 unique proteins were identified (Figure 2a). Details of all proteins are specified in Supplementary table 2.

To investigate cellular origins of SF EVs, expression of specific cellular lineage protein markers across individual patients was assessed (Figure 2b). Consistent with previous observations describing neutrophils as the major source of EVs in RA joints,^7^ neutrophil markers such as integrin alpha‐M, integrin beta‐2 and myeloperoxidase (MPO) were highly expressed in RA samples (Figure 2b). Fibroblast markers were also highly expressed, indicating FLS are also major producers of EVs in RA SF. Markers of T cell, DC, erythrocyte and endothelial cell origin were more prevalent in EVs obtained from RA joints with high‐level inflammation, indicating involvement of EVs from these cell types in highly inflamed rheumatoid joints. In contrast to previous reports describing high levels of B cell^9^ and platelet^5^‐derived EVs in RA SF, B cell or platelet markers were not detected. FLS and neutrophil markers were enriched in OA patients relative to other cellular markers, indicating these cells are a predominant source of EVs in OA SF.

### Citrullinated peptides are present in SF EVs of RA patients and are predicted to have greater affinity for HLA‐DR susceptibility alleles

Since citrullinated proteins can be recognised as autoantigens in RA and have previously been reported within SF EVs,^6^, ^13^ we investigated citrullinated peptides within our proteomics dataset. Nine citrullinated peptides were identified, all of which were only detected in EVs from RA joints with high‐level inflammation (Table 2). These included citrullinated peptides derived from fibrinogen alpha (FGA), actin [β‐actin (ACTB)/ACTG] and histone H3, which are known to be recognised by anti‐citrullinated peptide antibodies (ACPA).^14^, ^15^, ^16^ Consistent with this, the majority of patients, in whom citrullinated EV peptides were observed, were ACPA‐positive. MS spectra of the citrullinated peptides are presented in Supplementary figure 1.

**Table 2:** Citrullinated peptides identified in extracellular vesicles present in the synovial fluid of rheumatoid arthritis (RA) patients

Accession	Protein name	Citrullinated peptide sequence^a^	Mass‐to‐charge ratio (m/z)	Parts per million	Retention time	−10LogP (PEAKS)	Score (MQ)	Sample detected in/ACPA status
P16070|CD44_HUMAN	CD44 antigen	ESSETPDQFMTADET**citR**NLQNVDMK	929.7452	0.8	62.7	82.14	138.08	RA‐HIGH_2/ ACPA‐negative
P63261|ACTG_HUMAN: P60709|ACTB_HUMAN	Actin cytoplasmic 2	IWHHTFYNEL**citR**VAPEEHPVLLTEAPLNPK	691.1657	2.0	66.5	71.15	84.52	RA‐HIGH_2/ ACPA‐negative
P02671|FIBA_HUMAN	Fibrinogen alpha chain	GDFSSANN**citR**DNTYNR	577.9180	0.7	11.7	65.31	100.54	RA‐HIGH_5/ ACPA‐positive
P21730|C5AR1_HUMAN	C5a anaphylatoxin chemotactic receptor 1	SFT**citR**STVDTMAQK	491.5763	2.6	27.3	54.95	109.42	RA‐HIGH_2/ ACPA‐negative
Q92954|PRG4_HUMAN	Proteoglycan 4	AITT**citR**SGQTLSK	632.3525	2.1	8.7	48.71	NA	RA‐HIGH_5/ ACPA‐positive
P31146|COR1A_HUMAN	Coronin‐1A	**citR**AAPEASGTPSSDAVSR	830.4032	−0.2	13.6	48.01	NA	RA‐HIGH_2/ ACPA‐negative
P84095|RHOG_HUMAN	Rho‐related GTP‐binding protein RhoG	TVNLNLWDTAGQEEYD**citR**LR	765.7108	0.3	30.1	45.34	113.05	RA‐HIGH_2/ ACPA‐negative
Q71DI3|H32_HUMAN: P68431|H31_HUMAN: P84243|H33_HUMAN	Histone H3.2	YRPGTVAL**citR**EIR	477.9438	3.2	30.1	42.79	NA	RA‐HIGH_5/ ACPA‐positive
P02671|FIBA_HUMAN	Fibrinogen alpha chain	MELE**citR**PGGNEITR	501.5828	1.6	25.7	37.03	NA	RA‐HIGH_4/ ACPA‐positive

^a^ The position of citrullinated arginine residues are indicated in bold.

To investigate whether citrullination increases the likelihood of MHC‐II presentation, binding affinities of wild‐type and modified peptides for RA susceptibility HLA‐DR alleles were predicted using the online tool NetMHCII (Table 3). As NetMHCII does not permit input of citrulline residues, citrulline was interchanged with the amino acid glutamine, which has similar physicochemical properties. This approach was first validated on vimentin (VIM) and cartilage intermediate layer protein (CILP) peptides which are known to display greater binding affinities for certain HLA‐DR RA susceptibility alleles following citrullination.^17^, ^18^ Specifically, glutamine substitutions at position 71 of VIM and position 988 of CILP were predicted to improve affinity for HLA‐DR RA susceptibility alleles compared to the native peptides, confirming that glutamine affects MHC‐II binding in a similar manner to citrulline. Consistent with improved MHC‐II presentation of multiple citrullinated peptides identified within our proteomics dataset, replacement of arginine with glutamine at predicted sites of citrullination increased the predicted binding affinity to common RA HLA‐DR susceptibility alleles (Table 3). In particular, citrullination of rho‐related GTP‐binding protein RhoG (RHOG) at residue 66 was predicted to result in very strong binding to the DRB1*10:01 susceptibility allele.

**Table 3:** Binding affinity of WT and modified peptides to HLA class II rheumatoid arthritis susceptibility alleles^a^

	VIM 66–78		CILP 983–995		ACTG1 87–103		C5AR1 329–345		CD44 723–739		CORO1A 408–424		FGA 115–131		FGA 255–271		HIST2H3A 42–58		PRG4 1383–1399		RHOG 58–74	
Susceptibility allele	WT^b^	Mod	WT	Mod	WT	Mod	WT	Mod	WT	Mod	WT	Mod	WT	Mod	WT	Mod	WT	Mod	WT	Mod	WT	Mod
DRB1*01:01	27	**10**	143	**75**	642	**473**	> 1000	**925**	> 1000	> 1000	> 1000	> 1000	> 1000	> 1000	> 1000	> 1000	605	**457**	> 1000	**205**	> 1000	> 1000
DRB1*04:01	> 1000	**116**	> 1000	**474**	> 1000	> 1000	> 1000	> 1000	> 1000	> 1000	> 1000	> 1000	> 1000	> 1000	> 1000	> 1000	> 1000	> 1000	> 1000	> 1000	149	226
DRB1*04:04	34	**19**	43	49	956	> 1000	> 1000	> 1000	> 1000	> 1000	> 1000	> 1000	> 1000	> 1000	> 1000	> 1000	851	**482**	> 1000	> 1000	> 1000	> 1000
DRB1*04:05	> 1000	**408**	274	**202**	139	**87**	532	813	> 1000	**720**	> 1000	> 1000	> 1000	**323**	> 1000	> 1000	> 1000	> 1000	> 1000	> 1000	194	626
DRB1*10:01	467	**283**	> 1000	> 1000	82	**66**	493	**326**	274	329	> 1000	**664**	292	**120**	> 1000	**943**	372	**309**	564	**313**	42	**24**

^a^ Peptide sequences where x indicates position of modified arginine: VIM 66–78 (positive control), SAVRLxSSVPGVR; CILP 983–995 (positive control), KLYGIxDVRSTRD; ACTG1 87–103, HHTFYNELxVAPEEHPV; C5AR1 329–345, VRESKSFTxSTVDTMAQ; CD44 723–739, QFMTADETxNLQNVDMK; CORO1A 408–424, RGLDTGRRxAAPEASGT; FGA 115–131, GDFSSANNxDNTYNRVS; FGA 255–271, PQMRMELExPGGNEITR; HIST2H3A 42–58, YRPGTVALxEIRRYQKS; PRG4 1383–1399, RTARAITTxSGQTLSKV; RHOG 58–74, TAGQEEYDxLRTLSYPQ.

^b^ Predicted binding affinity expressed as half‐maximal inhibitory concentration (IC50) nm. Modified peptides with increased binding affinity are highlighted in bold. Weak binders with an IC50 > 1000 nm are indicated in grey.

### Fibrinogen‐β chain, immunoglobulins and annexins are prevalent in SF EVs from RA SF

Proteins expressed at high levels might provide insight into the contributions of EVs to RA. The 10 highest ranked proteins across all RA patients, and their potential functional roles, are listed in Table 4. Most of these proteins were present at higher levels in RA patients than OA patients and were further enriched across RA patients with high‐level inflammation. Highly ranked proteins include fibrinogen‐β chain (FGB) and ACTB, which are both recognised as autoantigens in RA following citrullination.^15^, ^19^ In addition, the immunoglobulin, Ig kappa chain constant region (IGKC), was highly ranked. Interestingly, anti‐inflammatory capabilities of SF EVs might be indicated by high levels of ANXA1, which has been reported to mediate beneficial effects of EVs derived from TNF‐stimulated neutrophils.^7^, ^8^

**Table 4:** Highest ranked synovial fluid extracellular vesicle (EV) proteins across all rheumatoid arthritis (RA) patients

Rank	UniProt gene	UniProt protein	RA ‐ all patients (avg. iBAQ)	RA high‐level inflammation (avg. iBAQ)	RA low‐level inflammation (avg. iBAQ)	OA (avg. iBAQ)	Potential involvement in RA/EV biology	Reference
1	ANXA2	Annexin A2	770 724	955 269	512 361	417 606	Potential autoantigen	Salle *et al*. 2008^52^
2	GLIPR2	Golgi‐associated plant pathogenesis‐related protein 1	713 566	954 927	375 660	53 732	Epithelial cell migration	Huang *et al*. 2013^53^
3	FGB	Fibrinogen beta chain	773 331	1 031 950	411 263	152 131	Defined autoantigen	Muller & Radic 2015^19^
4	ANXA1	Annexin A1	545 978	816 539	167 194	137 854	Cartilage regeneration	Headland *et al*. 2015^7^
5	MSN	Moesin	329 042	398 267	232 126	77 226	EV migration	Dalli *et al*. 2013^29^
5	ANXA4	Annexin A4	309 971	388 451	200 099	114 765	EV membrane integrity	Boye *et al*. 2017^54^
7	ACTB	Actin, cytoplasmic 1	409 856	396 346	428 769	132 114	Defined autoantigen	Darrah *et al*. 2012^15^
8	IGKC	Ig kappa chain C region	769 398	1 124 444	272 333	41 868	Adaptive immune response	Klareskog *et al*. 2014^55^
9	ARF1	ADP‐ribosylation factor 1	300 485	390 117	175 000	95 091	EV biogenesis	Record *et al*. 2018^56^
10	ANXA6	Annexin A6	276 676	355 757	165 962	90 493	EV membrane integrity	Boye *et al*. 2017^54^

iBAQ, intensity‐based absolute quantification.

### Immunogenic and pro‐inflammatory proteins within EVs from RA SF support pathogenic functionality

**Figure 3:**
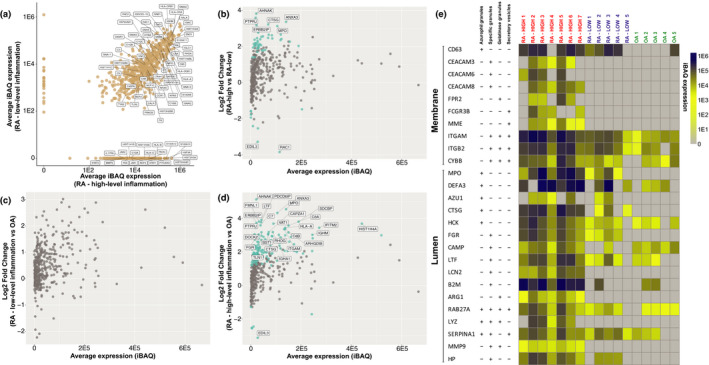
Pro‐inflammatory proteins are enriched in synovial fluid (SF) extracellular vesicles (EVs). **(a)** Scatter plot comparing average intensity‐based absolute quantification (iBAQ) protein expression between SF EVs rheumatoid arthritis (RA) patients with high‐ and low‐level inflammation. Selected proteins associated with RA pathology are labelled. **(b–d)** MA plots of protein abundance vs fold change comparing differences in protein expression between **(b)** RA (high‐level inflammation) vs RA (low‐level inflammation), **(c)** RA (low‐level inflammation) vs OA and **(d)** RA (high‐level inflammation) vs OA. 399 proteins met criteria for inclusion in the differential expression analysis and are represented as dots. Proteins with an *adj. P‐*value < 0.05 are highlighted in blue. Proteins with an *adj. P‐*value < 0.05 and a log_2_ fold change > 3 are labelled. **(e)** Expression of canonical neutrophil granule proteins detected in SF EVs as illustrated by heatmap. Heatmap columns represent individual SF EV samples and rows refer to corresponding granule proteins. Membrane or luminal location of granule proteins is indicated as well as the granule subset in which they are located. iBAQ values are scaled as indicated. UniProt gene names are specified. The table of granule proteins and respective locations is adapted from Cowland *et al*.^51^.

To further evaluate SF EVs in RA, we investigated known disease‐associated proteins within the proteomics dataset. A number of potent pro‐inflammatory drivers were identified (Figure 3a), including signal transducer and activator of transcription 1 (STAT1) and 3 (STAT3), tyrosine‐protein kinases JAK1, JAK2 and TYK2, apoptosis‐associated speck‐like protein containing a CARD (PYCARD), Toll‐like receptor 2 (TLR2), matrix metalloproteinase‐9 (MMP9), protein kinase C beta type (PRKCB), C‐C chemokine receptor type 1 (CCR1), tumor necrosis factor receptor superfamily member 6 (FAS) and leukotriene A‐4 hydrolase (LTA4H), as well as numerous S100 calcium‐binding, complement and ras family proteins. Additional proteins potentially recognised as autoantigens following citrullination were present, including calreticulin‐3 (CALR), VIM, F‐actin‐capping protein subunit alpha‐1 (CAPZA1), heat‐shock protein 90‐beta (HSP90AB1), alpha‐enolase (ENO1) and H1, H2 and H3 histone proteins.^19^ Protein‐arginine deiminase type‐4 (PADI4), numerous immunoglobulins and MHC‐II components, including HLA‐DRB1, were also detected, consistent with roles for EVs in autoantigen presentation. Proteins associated with cell proliferation and G protein signal transduction were present, including the GTPases: KRas, NRas, Ral‐A, RhoA, in addition to various G proteins and G protein‐coupled receptors. Furthermore, NADPH oxidase components: RAC1, RAC2, NCF4, NCF1C, NCF2 and cytochrome b‐245 light (CYBA) and heavy (CYBB) were all present, in addition to high MPO levels. Overall, these data suggest EVs within RA SF promote disease via a diverse cargo of immunogenic and inflammatory proteins. Additionally, many of these proteins were only detected in joints with high‐level inflammation, in keeping with the possibility that EVs can propagate disease (Figure 3a and Supplementary table 2).

### SF EV proteins are differentially expressed between joints with high‐ and low‐level inflammation

We next applied a label‐free quantitative MS approach to identify protein expression profile differences between SF EV samples from RA joints with high‐ and low‐level inflammation. Consistent with our gel electrophoresis observations showing greater protein diversity in SF EVs from joints with high‐level inflammation (Figure 1f), differential expression analysis of the proteomics dataset identified 45 proteins significantly increased in EVs from joints with high‐level inflammation, whereas only nine proteins were significantly increased in EVs from joints with low‐level inflammation (Figure 3b and Table 5). Gene ontology analysis was performed to investigate overrepresented biological processes associated with the 45 proteins increased in EVs from joints with high‐level inflammation. ‘Neutrophil degranulation’ was decisively the highest ranked biological process (*adj. P‐*value = 2.4E‐15, fold enrichment = 15) with 20/45 proteins associated with this pathway (Supplementary table 3), including MPO and cathepsin G (CTSG).

**Table 5:** Details of the 54 synovial fluid extracellular vesicle (EV) proteins significantly differentially expressed between RA patients with high and low‐level inflammation

Majority protein IDs	Protein names	Gene names	Log_2_ fold change (RA‐high vs RA‐low)	*P*‐value	adj. *P*‐value	RA high‐level inflammation (avg. iBAQ)	RA low‐level inflammation (avg. iBAQ)
Q12913	Receptor‐type tyrosine‐protein phosphatase eta	PTPRJ	3.59	9.89E‐09	3.94E‐06	25 935	526
P08311	Cathepsin G	CTSG	3.23	5.46E‐07	1.09E‐04	87 686	5070
P63000	Ras‐related C3 botulinum toxin substrate 1	RAC1	–3.84	4.89E‐06	5.23E‐04	54 423	117 295
P52907	F‐actin‐capping protein subunit alpha‐1	CAPZA1	2.66	5.24E‐06	5.23E‐04	76 412	20 530
Q09666	Neuroblast differentiation‐associated protein AHNAK	AHNAK	3.82	8.90E‐06	7.10E‐04	28 049	1375
Q9H3M7	Thioredoxin‐interacting protein	TXNIP	2.88	1.14E‐05	7.61E‐04	52 600	5945
P62993	Growth factor receptor‐bound protein 2	GRB2	2.13	1.73E‐05	9.84E‐04	44 316	2895
Q15833	Syntaxin‐binding protein 2	STXBP2	2.77	2.64E‐05	1.32E‐03	38 532	922
P31146	Coronin‐1A	CORO1A	2.19	3.77E‐05	1.54E‐03	23 667	200
O43854	EGF‐like repeat and discoidin I‐like domain‐containing protein 3	EDIL3	–3.27	3.85E‐05	1.54E‐03	1850	36 873
Q9BZQ8	Protein Niban	FAM129A	2.47	5.78E‐05	2.10E‐03	30 354	2144
Q96RT1	Protein LAP2	ERBB2IP	3.18	1.31E‐04	4.35E‐03	24 093	200
P22748	Carbonic anhydrase 4	CA4	2.25	1.74E‐04	5.35E‐03	36 457	200
P12429	Annexin A3	ANXA3	3.37	1.95E‐04	5.51E‐03	181 783	3022
P23381	Tryptophan‐‐tRNA ligase, cytoplasmic	WARS	2.31	2.07E‐04	5.51E‐03	35 910	200
Q9H4M9	EH domain‐containing protein 1	EHD1	2.66	3.87E‐04	9.66E‐03	141 723	14 839
Q01518	Adenylyl cyclase‐associated protein 1	CAP1	1.91	4.48E‐04	1.01E‐02	35 811	3355
P34910	Protein EVI2B	EVI2B	2.56	4.69E‐04	1.01E‐02	62 321	1024
P43250	G protein‐coupled receptor kinase 6	GRK6	2.15	5.04E‐04	1.01E‐02	27 528	2276
O43795	Unconventional myosin‐Ib	MYO1B	–1.77	5.11E‐04	1.01E‐02	477	1742
Q14254	Flotillin‐2	FLOT2	2.04	5.65E‐04	1.01E‐02	25 735	200
P04114	Apolipoprotein B‐100	APOB	–1.93	5.74E‐04	1.01E‐02	404	1208
O75340	Programmed cell death protein 6	PDCD6	1.81	5.98E‐04	1.01E‐02	75 784	200
P15144	Aminopeptidase N	ANPEP	2.15	6.50E‐04	1.01E‐02	14 992	404
Q10588	ADP‐ribosyl cyclase/cyclic ADP‐ribose hydrolase 2	BST1	2.48	6.51E‐04	1.01E‐02	89 336	1685
P20701	Integrin alpha‐L	ITGAL	2.11	6.60E‐04	1.01E‐02	37 548	4250
Q8WUM4	Programmed cell death 6‐interacting protein	PDCD6IP	2.07	6.95E‐04	1.03E‐02	50 966	4219
P22681	E3 ubiquitin‐protein ligase CBL	CBL	–1.57	1.10E‐03	1.57E‐02	1339	200
P09769	Tyrosine‐protein kinase Fgr	FGR	1.94	1.19E‐03	1.64E‐02	39 361	3112
P13796	Plastin‐2	LCP1	2.62	1.30E‐03	1.73E‐02	102 681	7650
O94804	Serine/threonine‐protein kinase 10	STK10	2.04	1.40E‐03	1.80E‐02	18 346	1462
P08631	Tyrosine‐protein kinase HCK	HCK	2.40	1.51E‐03	1.85E‐02	46 555	3095
Q92608	Dedicator of cytokinesis protein 2	DOCK2	2.38	1.53E‐03	1.85E‐02	19 353	1357
P15924	Desmoplakin	DSP	–1.58	1.77E‐03	2.08E‐02	565	567
P62879	Guanine nucleotide‐binding protein G(I)/G(S)/G(T) subunit beta‐2	GNB2	2.42	2.06E‐03	2.35E‐02	114 852	52 859
P05164	Myeloperoxidase	MPO	3.13	2.30E‐03	2.51E‐02	130 125	11 933
P62937	Peptidyl‐prolyl cis‐trans isomerase A	PPIA	2.27	2.38E‐03	2.51E‐02	168 691	34 411
P02788	Lactotransferrin	LTF	2.54	2.44E‐03	2.51E‐02	49 784	7385
P52209	6‐phosphogluconate dehydrogenase, decarboxylating	PGD	1.71	2.45E‐03	2.51E‐02	30 425	5364
O14745	Na^+^/H^+^ exchange regulatory cofactor NHE‐RF1	SLC9A3R1	1.88	2.67E‐03	2.66E‐02	32 871	200
P61225	Ras‐related protein Rap‐2b	RAP2B	1.80	2.79E‐03	2.71E‐02	63 501	3820
P01011	Alpha‐1‐antichymotrypsin	SERPINA3	2.17	3.87E‐03	3.68E‐02	18 510	717
P29350	Tyrosine‐protein phosphatase non‐receptor type 6	PTPN6	2.06	4.07E‐03	3.73E‐02	38 283	9891
P16112	Aggrecan core protein	ACAN	–2.53	4.15E‐03	3.73E‐02	9688	33 045
P02730	Band 3 anion transport protein	SLC4A1	–2.40	4.20E‐03	3.73E‐02	1705	14 759
O00186	Syntaxin‐binding protein 3	STXBP3	2.03	4.30E‐03	3.73E‐02	30 413	7517
P00450	Ceruloplasmin	CP	1.95	4.58E‐03	3.88E‐02	12 684	200
Q86YV0	RAS protein activator like‐3	RASAL3	1.40	4.73E‐03	3.93E‐02	9506	200
A8MVU1	Putative neutrophil cytosol factor 1C	NCF1C	1.42	4.90E‐03	3.99E‐02	17 988	2835
Q08431	Lactadherin	MFGE8	–1.47	5.32E‐03	4.25E‐02	98 858	173 025
O00560	Syntenin‐1	SDCBP	2.03	5.43E‐03	4.25E‐02	229 002	23 613
P00734	Prothrombin	F2	2.45	5.59E‐03	4.29E‐02	102 582	12 151
Q9NUQ9	Protein FAM49B	FAM49B	2.36	6.49E‐03	4.89E‐02	137 390	14 926
P0DMV9	Heat‐shock 70‐kDa protein 1B	HSPA1B	1.78	6.65E‐03	4.91E‐02	53 304	5265

iBAQ, intensity‐based absolute quantification; RA, rheumatoid arthritis.

Nine proteins were significantly enriched in EVs from joints with low‐level inflammation (Table 5 and Supplementary table 2). However, gene ontology analysis of these proteins did not reveal any significantly overrepresented biological processes (data not shown). Notably, the signal transducing protein Ras‐related C3 botulinum toxin substrate 1 (RAC1) was markedly increased (14‐fold) in low‐level inflammation. Given RAC1 is associated with FLS proliferation and invasion,^20^ and T‐cell activation,^21^ these data suggest that even in rheumatoid joints with low‐level inflammation, SF EVs may have destructive potential.

### SF EV proteins are differentially expressed between OA and RA joints with high‐level inflammation

We next compared differences in SF EV protein content between RA and OA joints. While no SF EV proteins were significantly differentially expressed between RA joints with low‐level inflammation and OA joints (Figure 3c and Supplementary table 2), 135 proteins were significantly enriched in SF EVs from the highly inflamed joints of RA patients, and 6 proteins were significantly enriched in OA joints (Figure 3d and Supplementary table 2). To investigate biological pathways associated with the 135 proteins increased in the joints of RA patients with high‐level inflammation, gene ontology was performed. ‘Neutrophil degranulation’ was again the highest ranked biological process (*adj. P‐*value = 8.0E‐28, fold enrichment = 10) with 42/135 proteins associated with this pathway (Supplementary table 4). Enrichment for proteins associated with platelet aggregation (*adj. P‐*value = 4.6E‐9), complement activation (*adj.P‐*value = 1.2E‐6) and leucocyte migration (*adj. P*‐value = 1.7E‐5) was also apparent. The 6 proteins significantly enriched in SF EVs from OA joints were as follows: EGF‐like repeat and discoidin I‐like domain‐containing protein 3 (EDIL3), prosaposin (PSAP), mannan‐binding lectin serine protease 2 (MASP2), nucleophosmin (NPM1), keratin 2 (KRT2) and myosin‐Ib (MYO1B).

### SF EVs in the joints of RA patients with high‐level inflammation are enriched for neutrophil granule proteins

Because neutrophils were identified as major producers of SF EVs (Figure 2b), and proteins associated with neutrophil degranulation were enriched in EVs from RA joints with high‐level inflammation (Supplementary tables 3 and 4), expression of specific granule proteins in the proteomics dataset was further investigated. Membrane and luminal proteins from azurophilic, specific, gelatinase granules and secretory vesicles were detected in both RA subgroups and at greater levels in EVs from joints with high‐level inflammation (Figure 3e).

## Discussion

Using a SEC method for high‐quality EV enrichments, we show that SF EVs from RA patients contain a protein cargo with the capacity to modulate immune and inflammatory responses. These results provide new insight into how EVs may regulate local inflammatory processes in synovial joints and thereby contribute to the perpetuation of RA.

The presence of citrullinated proteins and high levels of immunoglobulins in RA SF EVs is consistent with previous observations that SF EVs contain citrullinated autoantigens that facilitate formation of pro‐inflammatory immune complexes.^6^, ^13^ We detected citrullinated peptides derived from FGA and proteoglycan 4 in RA patients positive for ACPAs, supporting immunogenic roles for citrullinated forms of these proteins in RA,^22^ as well as potential involvement of EVs in generating immune responses.^6^ We also identified citrullinated peptides not previously described in RA that might represent novel autoantigens. In particular, citrullination of RHOG at position 66 is predicted to result in high affinity for HLA‐DRB1*10:01. A number of additional autoantigenic proteins were also identified, including high levels of FGB. Citrullinated FGB may associate with SF EVs in RA, but might not have been detected because proteinase K was used to deplete non‐EV‐associated material.

Our MS data revealed a number of interesting proteins that might explain previously reported pro‐inflammatory effects of EVs from RA SF.^3^, ^4^, ^5^, ^6^ For instance, EV‐encapsulated STAT and JAK proteins might facilitate chemokine and cytokine release from FLS exposed to RA SF EVs.^3^ Other notable EV proteins possibly contributing to inflammatory responses in recipient cells include PYCARD, TLR2, PRKCB, CCR1, FAS and LTA4H.

The 54 proteins differentially expressed between RA joints with high‐ and low‐level inflammation provide strong candidates for further investigations into SF EV proteins that regulate inflammatory processes in RA. Notably, CTSG was increased 10‐fold in joints with high‐level inflammation (*adj.P*‐value = 1.1E4) and is of specific interest given its roles in chemokine activation and autoantigen processing.^23^ RAC1 was increased by 14‐fold in joints with low‐level inflammation (*adj. P*‐value = 5.2E4). RAC1 has been identified as a potential therapeutic target in RA^21^ and has roles in inflammation and immune responses – including through T‐cell^21^ and FLS activation,^20^ ROS generation^24^ and facilitating effective antigen presentation in DCs.^25^ Future studies to determine whether SF EV‐associated CTSG and RAC1 are involved in pathological pathways in RA would be of interest.

Rheumatoid arthritis SF EVs were highly enriched for neutrophil lineage markers and neutrophil granule proteins, including MPO, which was ninefold increased in RA joints with high‐level inflammation than in low‐level inflammation (*adj. P‐*value = 0.025). MPO is a highly reactive neutrophil azurophilic granule protein that has pathological roles in RA. High levels of MPO are found in RA synovium where it promotes oxidative stress through HClO production^26^ and FLS expansion^27^ and is required for formation of neutrophil extracellular traps during NETosis.^28^ Consistent with this, MPO deficiency reduces disease severity in K/BxN and CIA murine models of inflammatory arthritis.^27^ High levels of MPO have previously been identified in neutrophil‐derived EVs,^29^, ^30^, ^31^, ^32^ although our study is the first to show specific enrichment of MPO in RA SF EVs. Consistent with destructive effects for EV‐associated MPO, EVs derived from calcium ionophore‐stimulated neutrophils mediated endothelial cell damage in an MPO‐dependent manner.^33^ EVs from latrunculin B + N‐formylmethionyl‐leucyl‐phenylalanine stimulated neutrophils were shown to inhibit epithelial cell migration, proliferation and healing of intestinal epithelium through MPO.^32^ Although we have not formally demonstrated functional activity of EV‐associated MPO, it is conceivable that SF EVs might help to mediate MPO's destructive effects in RA.

In some circumstances, neutrophil‐derived EVs might also mediate anti‐inflammatory effects. For example, neutrophil‐derived EVs have been reported to inhibit T‐cell proliferation,^34^ DC maturation^35^ and pro‐inflammatory cytokine release from NK cells.^36^ EVs derived from TNF‐stimulated peripheral blood neutrophils from healthy donors might also promote synthesis of components involved in cartilage regeneration, mediated through ANXA1.^7^ EVs derived from TNF‐stimulated healthy and RA peripheral blood neutrophils were also shown to promote anti‐inflammatory effects in recipient macrophages via ANXA1.^8^ In our study, ANXA1 was the fourth most prevalent protein detected in SF EVs and was mildly increased 1.5‐fold in RA joints with high‐level inflammation than in low‐level inflammation albeit not in a statistically significant manner (*adj. P‐*value = 0.22). Nevertheless, it is thus conceivable that neutrophil‐derived EVs present in RA SF may have protective effects mediated through ANXA1. However, the *in vitro* stimuli used to generate the EVs in the above studies may not reflect the complex microenvironment of the inflamed joint *in vivo*. Functional effects from neutrophil‐derived EVs may depend on the state of the originating neutrophil and the stimuli employed. For example, incubation of endothelial cells with EVs generated from suspended neutrophils promoted pro‐inflammatory gene expression, whereas cells incubated with EVs from adherent neutrophils induced anti‐inflammatory gene expression.^29^ Furthermore, different stimuli can affect the concentration and size of neutrophil EVs.^37^ Thus, it is conceivable that EVs arising *in vivo* from differentially activated neutrophils, neutrophils undergoing apoptosis and neutrophils undergoing necrotic cell death may vary. For example, degranulating neutrophils might release smaller EVs enriched with stimulatory granule proteins. Further assessment of the factors that affect neutrophil‐derived EV content and function in RA is of great interest.

Proteins enriched in EVs in SF of patients with OA might also have both protective and pathogenic effects. Compared to RA joints with high‐level inflammation, EGF‐like repeat and discoidin I‐like domain‐containing protein 3 (EDIL3) was ninefold increased in OA SF EVs (*adj. P‐*value = 0.001). Given deletion of EDIL3 is associated with increased chondrocyte apoptosis and more severe OA,^38^, ^39^ this increase in EDIL3 in OA joints might thus be protective. EDIL3 was similarly enriched in SF EVs from RA joints with low‐level inflammation, than in RA joints with high‐level inflammation (10‐fold; *adj. P‐*value = 0.002), suggesting EDIL3 might also have chondroprotective effects in RA. MASP‐2, on the other hand, was sixfold increased in OA SF EVs compared to EVs from RA joints with high‐level inflammation (*adj. P‐*value = 0.005) and would be consistent with OA SF EVs exerting a pathogenic effect by promoting complement activation in OA.^40^

Our identification of neutrophils and FLS as the major source of EVs in RA SF is in contrast to previous studies which suggested B cells^9^ and platelets^5^ as major sources. Interestingly, in our study neither B cell nor platelet lineage markers were detected. Variations in sample handling and EV isolation procedures may have contributed to these differences.^1^ For instance, as the cell lineage markers used in our study to phenotype EVs are membrane bound, the use of proteinase K to deplete non‐EV material may have removed markers of B cell and platelet origin, making these harder to detect by MS and thus potentially obscuring the contribution of these cells to RA SF EVs. This might also explain why the CD3 T‐cell protein was detected, but CD4 and CD8 were not. SF EVs most likely reflect the proportions of corresponding cell types in the SF. For example, elevated SF neutrophils in RA SF presumably contribute to high numbers of neutrophil‐derived EVs. However, we were unable to profile leucocyte subsets in the SF of our current cohort to test this hypothesis, and subsequent studies investigating correlations between SF cell and EV subtypes will be of interest.

Finally, although an increasing body of literature describes immunomodulatory functions for EVs, including SF EVs, few studies have investigated functional effects of RA SF EVs on recipient synovial and immune cells *in vivo*. SF EVs could directly stimulate responses in adjacent FLS and synovial macrophages. These responses may be cell‐specific, as synovial macrophages might phagocytose EVs more efficiently compared to synovial fibroblasts. Determining the functional potency of EVs within inflamed synovial joints and how EV cargo changes at different stages of joint disease are exciting avenues for future research.

## Methods

We have submitted all relevant data of our experiments to the EV‐TRACK knowledgebase (EV‐TRACK ID: EV190097).^41^

### Patient details, collection and storage of human synovial fluid

Synovial fluid was obtained from RA patients undergoing arthrocentesis as previously described^11^ and used with informed consent and the approval of the Melbourne Health Research and Ethics Committee (project nos 2005.056 and 2010.293). SF was centrifuged at 2000 *g* for 20 min to remove cells, then aliquoted and stored at −80°C until the time of experimentation. SF was classified as originating from RA joints with either high‐ or low‐level inflammation by the treating rheumatologist on the basis of several criteria. Specifically, the inflammatory status of the index aspirated joint was assessed based on the presence of SF white cell counts of either greater or < 2000 cells µL^−1^ (note these are all either > 4000 cells µL^−1^ or < 1000 cells µL^−1^) – this is a ‘traditional’ cut‐off, assessed as having reasonable diagnostic performance characteristics (sensitivity, 0.84; specificity, 0.84).^12^ Cell counts were performed using standard microscopy techniques in the Royal Melbourne Hospital's Pathology service, as part of routine clinical care. Unfortunately, no further subtyping of SF white cells is available. Patient demographics and clinical parameters are specified in Table 1 and Supplementary table 1.

### Sample preparation and EV isolation

Two to five milliliter of cell‐depleted SF was thawed and treated with hyaluronidase (Sigma‐Aldrich, North Ryde, NSW, Australia) at 30 U mL^−1^ and DNase I (Worthington Biochemical, Lakewood, CA, USA) at 20 U mL^−1^ for 15 min at 37°C. Enzyme‐treated, cell‐depleted SF was diluted to 13 mL with 4.84 mm EDTA/DPBS and centrifuged at 10 000 *g* (avg; 11 700 RPM, *k*‐Factor = 1563) for 30 min at 4°C in a 70 Ti rotor using polycarbonate tubes (Beckman Coulter, Mount Waverley, VIC, Australia). The supernatant was collected and injected into a HiPrep 26/60 Sephacryl S‐500 HR prepacked gel filtration column and eluted with 4.84 mm EDTA/DPBS at a flow rate of 1.5 mL min^−1^. SEC eluent from 60 to 120 min was collected, transferred to polycarbonate tubes and ultracentrifuged at 100 000 *g* (avg; 36 900 RPM, *k*‐Factor = 157) in a 70 Ti rotor for 90 min at 4°C to concentrate EVs. EV pellets were resuspended in 1 mL of DPBS and incubated at 37°C with proteinase K (Roche, Sydney, NSW, Australia) at 75 U mL^−1^. Phenylmethylsulfonyl fluoride was added at 0.625 mm to inactivate proteinase K and the sample ultracentrifuged at 58 100 *g* (avg; 35 900 RPM, *k*‐Factor = 157) for 90 min at 4°C in a polypropylene tube, using a TLA45 rotor. EV pellets were resuspended in lysis buffer (150 mm NaCl, 50 mm Tris at pH 7.4, 1% (v v^−1^) Triton X‐100, 0.5% (w v^−1^) sodium deoxycholate), containing a protease inhibitor cocktail (Roche), and stored at −80°C, or in DPBS for transmission electron microscopy. Protein content was measured by Pierce BCA Protein Assay (Thermo Fisher, Scoresby, VIC, Australia).

### Transmission electron microscopy

Proteinase K‐treated EVs in DPBS were fixed overnight at 4°C with 1% glutaraldehyde and adsorbed onto glow‐discharged 200 mesh formvar with carbon coating Cu grids (ProSciTech, Kirwan, QLD, Australia). Grids were washed twice with milliQ water, negatively stained with 2% uranyl acetate and imaged using a Talos L120C electron microscope (FEI, Hillsboro, OR, USA).

### Nanoparticle tracking analysis

Unconcentrated EV‐containing SEC eluent was collected from SF samples at the time of EV isolation and stored at −80°C. At the time of analysis, samples were thawed on ice and particle size and concentration were assessed on a NanoSight NS300 (Malvern Instruments, Malvern, UK). Each sample was analysed with the camera level, slider shutter, gain and syringe pump speed, respectively, set to 13, 1232, 219 and 50. For analysis, a detection threshold of 5 was applied and minimum track length set to auto. Five replicate videos of 30 s duration per sample were collected and results averaged. Analysis was performed with NTA 3.2 Dev Build 3.2.16 (Malvern Panalytical Ltd, Malvern, UK).

### Gel electrophoresis and Western blot analysis

Gel electrophoresis and Western blot analysis were performed on proteinase K‐treated samples as previously described.^11^ One microgram of protein per sample was loaded for gel electrophoresis with silver staining. Following electrophoresis, gels were fixed with 30% EtOH and 10% AcOH for 60 min and then incubated with 30% EtOH, 0.4 m NaAc and 0.2% Na_2_S_2_O_3_ for 90 min. Treated gels were washed three times for 10–15 min in H_2_O and incubated with 0.1% AgNO_3_ and 0.02% formaldehyde for 30 min. Gels were developed with 2.5% NaCO_3_ and 0.01% formaldehyde, washed and imaged on a GS‐900 Calibrated Densitometer (Bio‐Rad, Hercules, CA, USA) using Image Lab 6.0 software (Bio‐Rad).

### Protein digestion

Extracellular vesicles lysates were prepared for MS analysis as described previously,^42^ whereby lysates were simultaneously reduced and alkylated with 10 mm TCEP and 5.5 mm 2‐chloroacetamide for 10 min at 95°C. Carboxylate bead stock (4 µL) and acetonitrile were added to a final concentration of 70% (v v^−1^). Beads were left to precipitate for 20 min at room temperature and then washed twice with 70% EtOH and once with acetonitrile. Beads were transferred to a 96‐well plate and acetonitrile was completely evaporated from the sample prior to the addition of 40 µL digestion buffer (10% 2‐2‐2‐trifluoroethanol, 100 mm NH_4_HCO_3_) containing 1 µg Trypsin‐gold (Promega, Madison, WI, USA). The plate was briefly sonicated in a water bath to disperse the beads and then transferred to a ThermoMixer instrument for digestion at 37°C for 60 min (600 RPM). The supernatant comprising peptides was then collected from the beads using a magnetic rack. An additional elution with 50 µL of 2% dimethyl sulfoxide (Sigma‐Aldrich) was performed on the beads. The peptides were desalted on in‐house made C18 stage tips (2x plugs of 3M Empore resin, #2215) and lyophilised to dryness using a CentriVap (Labconco, Kansas City, MO, USA), prior to reconstituting in 15 µL 0.1% formic acid and 2% acetonitrile for MS analysis.

### Mass spectrometry analysis

Peptides (3 µL) were separated by reverse‐phase chromatography on a C18‐fused silica column (I.D. 75 µm, O.D. 360 μm × 25 cm length) packed into an emitter tip (IonOpticks, Middle Camberwell, VIC, Australia), using a nano‐flow HPLC (M‐class; Waters, Wimslow, UK). The HPLC was coupled to an Impact II UHR‐QqTOF mass spectrometer (Bruker, Billerica, MA, USA) using a CaptiveSpray source and nanoBooster at 0.20 Bar using acetonitrile. Peptides were loaded directly onto the column at a constant flow rate of 400 nL min^−1^ with 0.1% formic acid in milliQ water and eluted with a 90 min linear gradient from 2 to 34% (99.9% acetonitrile and 0.1% formic acid). Mass spectra were acquired in a data‐dependent manner including an automatic switch between MS and MS/MS scans using a 1.5‐s duty cycle and 4 Hz MS1 spectra rate, followed by MS/MS scans at 8–20 Hz dependent on precursor intensity for the remainder of the cycle. MS spectra were acquired between a mass range of 200–2000 *m*/*z*. Peptide fragmentation was performed using collision‐induced dissociation.

Raw files consisting of high‐resolution MS/MS spectra were processed with MaxQuant (version 1.5.8.3) for feature detection and protein identification using the Andromeda search engine.^43^ Extracted peak lists were searched against the reviewed *Homo sapiens* database (UniProt, October 2016), as well as a separate reverse decoy database to empirically assess the false discovery rate (FDR) using strict trypsin specificity, allowing up to two missed cleavages. The minimum required peptide length was set to seven amino acids. In the main search, precursor mass tolerance was 0.006 Da and fragment mass tolerance was 40 ppm. The search included variable modifications of oxidation (methionine), amino‐terminal acetylation, the addition of pyroglutamate (at N‐termini of glutamine), deimination (R), deamidation (N/Q) and a fixed modification of carbamidomethyl (cysteine). The neutral loss of isocyanic acid (HNCO, 43.0058 Da) was added to the definition of citrullination (deimination, R) in the search algorithm. The ‘match between runs’ option in MaxQuant was used to transfer identifications made between runs on the basis of matching precursors with high mass accuracy.^44^, ^45^ LFQ quantification was selected, with a minimum ratio count of 2. Peptide‐spectrum matches (PSM) and protein identifications were filtered using a target‐decoy approach at an FDR of 1%. Only unique and razor peptides were considered for quantification with intensity values present in at least two out of three replicates per group. Statistical analyses were performed using LFQAnalyst^46^ (https://bioinformatics.erc.monash.edu/apps/LFQ‐Analyst/) whereby the LFQ intensity values were used for protein quantification. Missing values were replaced by values drawn from a normal distribution of 1.8 standard deviations and a width of 0.3 for each sample (Perseus‐type). Protein‐wise linear models combined with empirical Bayesian statistics were used for differential expression analysis using Bioconductor package limma, whereby the adjusted *P*‐value cut‐off was set at 0.05 and log_2_ fold change cut‐off set at 1. The Benjamini–Hochberg method of FDR correction was used. Raw MS data were also searched with PEAKS, version 8 (Bioinformatics Solutions, Waterloo, ON, Canada) using a Swiss‐Prot Human database and the same variable and fixed modifications as described above. A 0.1% and 1% FDR cut‐off was applied at the PSM and peptide/protein levels, respectively. MS/MS spectra were inspected manually to confirm citrullinated spectra.

The MS proteomics data have been deposited to the ProteomeXchange Consortium via the PRIDE^47^ partner repository with the dataset identifier: PXD015145.

A total of 1058 proteins were identified. Protein abundance was calculated using the intensity‐based absolute quantification (iBAQ) metric implemented in MaxQuant.^48^ iBAQ values are derived from the summed intensities of the precursor peptides that map to each protein and divided by the number of theoretically observable peptides. Therefore, iBAQ values are proportional to the molar quantities of proteins in a sample, which can be used to estimate the relative abundance of the proteins within each sample. Protein expression of lineage and granule markers is visualised with the pheatmap R package.

### Ranking of prevalent protein

To identify prevalent proteins within RA SF EVs, proteins were ranked in individual patients according to iBAQ intensity levels. For each protein, an average rank across all patients within a group (RA high inflammation/RA low inflammation) was calculated. Group ranks were then averaged, and proteins were given a final ranking according to the average group ranking. This method was selected to avoid undue influence of outliers and highly variable proteins that might confound average expression counts.

### Predicted binding affinity of modified peptides

Binding affinity of wild‐type and modified peptides was predicted with NetMHCII‐2.3.^49^ Arginine residues predicted to undergo citrullination and 8 flanking amino acids were input to NetMHCII‐2.3 as query sequences. As a result of the inability to input citrulline, deiminated arginine was represented as glutamine. Binding affinity was expressed as half‐maximal inhibitory concentration (IC50).

### Gene ontology analysis

Gene ontology analyses were performed with FunRich v3.1.3 using the UniProt database.^50^

## Author Contributions

**Andrew David Foers:** Conceptualization; Data curation; Formal analysis; Investigation; Methodology; Validation; Visualization; Writing‐original draft; Writing‐review & editing. **Laura Dagley:** Data curation; Formal analysis; Investigation; Methodology; Writing‐review & editing. **Simon Chatfield:** Conceptualization; Data curation; Resources. **Andrew Webb:** Methodology; Supervision. **Lesley Cheng:** Conceptualization; Formal analysis; Investigation; Methodology; Supervision; Writing‐review & editing. **Andrew F Hill:** Conceptualization; Formal analysis; Investigation; Methodology; Supervision; Writing‐review & editing. **Ian Wicks:** Conceptualization; Data curation; Formal analysis; Funding acquisition; Investigation; Methodology; Project administration; Resources; Supervision; Validation; Visualization; Writing‐original draft; Writing‐review & editing. **Kenneth Pang:** Conceptualization; Data curation; Formal analysis; Funding acquisition; Investigation; Methodology; Project administration; Resources; Supervision; Validation; Visualization; Writing‐original draft; Writing‐review & editing.

## Conflict of Interest

The authors declare no conflict of interest.
