# Supplementary material for: Proteomic analysis of extracellular vesicles reveals an immunogenic cargo in rheumatoid arthritis synovial fluid
Source: Clin Transl Immunology. 2020 Nov 7;9(11):e1185. doi: 10.1002/cti2.1185 (PMC7648259; doi:10.1002/cti2.1185)
Supplement: Supplementary file 1 [file CTI2-9-e1185-s001.pdf]

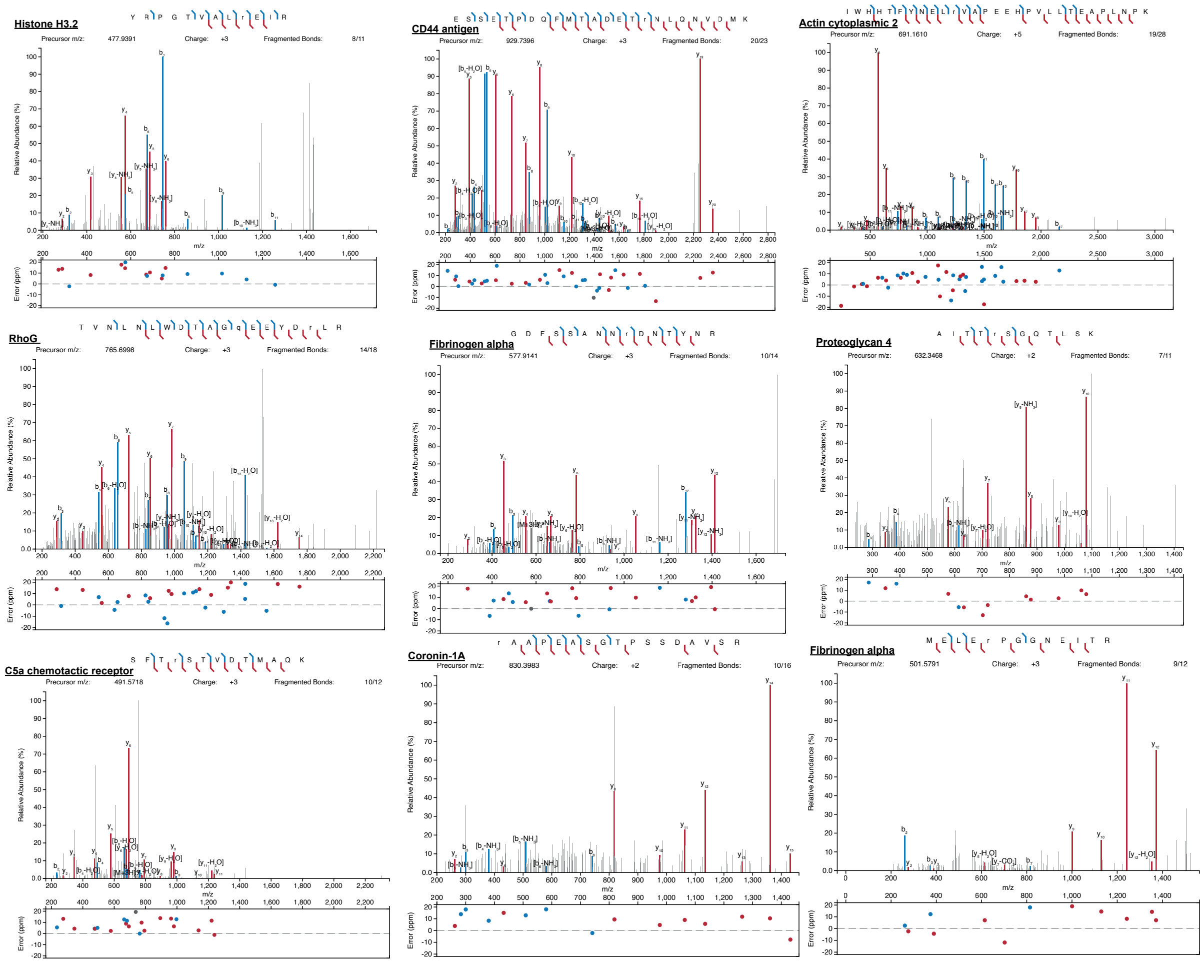

Supplementary figure 1: Mass spectra of citrullinated peptides

MS/MS spectra of peptides containing a citrulline (r) residue.
